# Construct Validity of the Upper-Limb Interlimb Coordination Test in Stroke

**DOI:** 10.1177/15459683211058092

**Published:** 2021-10-29

**Authors:** Roni Molad, Mindy F. Levin

**Affiliations:** 1 School of Physical and Occupational Therapy, 5620 McGill University, Montreal, QC, Canada; 2 Feil and Oberfeld Research Centre, 60387 Jewish Rehabilitation Hospital Site of Centre for Interdisciplinary Research in Rehabilitation, Montreal, QC, Canada

**Keywords:** motor coordination, stroke, kinematics, assessment, bimanual

## Abstract

**Background:**

Coordination impairments are under-evaluated in patients with stroke due to the lack of validated assessments resulting in an unclear relationship between coordination deficits and functional limitations.

**Objective:**

Determine the construct validity of the new clinical upper-limb (UL) Interlimb Coordination test (ILC2) in individuals with chronic stroke.

**Methods:**

Thirteen individuals with stroke, ≥40 years, with ≥30° isolated supination of the more-affected (MAff) arm, who could understand instructions and 13 healthy controls of similar age participated in a cross-sectional study. Participants performed synchronous bilateral anti-phase forearm rotations for 10 seconds in 4 conditions: self-paced internally-paced (IP1), fast internally-paced (IP2), slow externally-paced (EP1), and fast externally-paced (EP2). Primary (continuous relative phase-CRP, cross-correlation, lag) and secondary outcome measures (UL and trunk kinematics) were compared between groups.

**Results:**

Participants with stroke made slower UL movements than controls in all conditions, except EP1. Cross-correlation coefficients were lower (i.e., closer to 0) in stroke in IP1, but CRP and lag were similar between groups. In IP1 and matched-speed conditions (IP1 for healthy and IP2 for stroke), stroke participants used compensatory trunk and shoulder movements. The synchronicity sub-scale and total scores of ILC2 were related to temporal coordination in IP2. Interlimb Coordination test total score was related to greater shoulder rotation of the MAff arm. Interlimb Coordination test scores were not related to clinical scores.

**Conclusion:**

Interlimb Coordination test is a valid clinical measure that may be used to objectively assess UL interlimb coordination in individuals with chronic stroke. Further reliability testing is needed to determine the clinical utility of the scale.

## Introduction

Motor impairments are common following stroke and may result in limitations in the performance of daily activities, participation and quality of life.^1,2^ Performance limitations may be related to impairments in interlimb coordination, an important aspect of skilled arm use^3-5^ affected by stroke. However, this concept is neither well-defined nor well-measured clinically. This may be due to the focus of rehabilitation on improving unilateral performance, based on the assumption that reduced bimanual arm use results from deficits in the more-impaired upper-limb (UL).^6^ However, principles of interlimb coordination cannot be inferred from those of single-limb movements.^7-9^ Complex interactions between task requirements, environmental constraints, lesion location, level of sensorimotor impairment, and interhemispheric connectivity may all affect bimanual task accomplishment following stroke.^10,11^ The lack of consensus regarding the clinical definition of interlimb coordination makes it difficult to characterize. This has led to a poor understanding of the relationship between interlimb coordination deficits and limitations in functional recovery in stroke survivors.

Various perspectives on how the motor system controls and selects specific movement patterns despite its redundancy have been described, proposing different definitions of interlimb coordination.^12-15^ In particular, the dynamical systems theory suggests that coordination arises from the entrainment of dynamically coupled oscillators, reducing the number of possible solutions to a motor task. The concept of dynamical coupling has been implemented in a recent operational definition of coordination for reaching and pointing as “a goal-oriented and context-dependent process of organizing movements in both space and time.”^16^ Although originally formulated for intralimb coordination, this definition is also applicable to bimanual and bilateral movements.

Clinical UL interlimb coordination assessments at the International Classification of Functioning (ICF) Body Function and Structure level^17^ include rhythmic and discrete hand movements made at different frequencies, spatio-temporal relationships (e.g., timing, direction, and phase relationships) or with different loads,^18-21^ but their measurement properties have not been evaluated. At the ICF Activity level, assessments evaluating bimanual tasks, such as the Arm Motor Ability Test,^22^ rate task completion without objective description of how the task was performed and whether motor compensations were used.^23^ To provide a comprehensive description of coordinated movements that can distinguish motor recovery from compensation, movement should be described at 2 levels: performance and movement quality.^24^ The performance level describes the endpoint (i.e.,, hand) behavior using temporal and spatial variables. Movement quality refers to the joint rotations and displacements as well as motor compensations contributing to endpoint movement.^24^

The Comprehensive Coordination Scale (CCS) is a newly-developed objective outcome measure of the coordination of multiple body segments at both levels of movement description in people with neurological injuries.^25^ The CCS relies on observational kinematics for movement assessment and has 3 subscales with excellent intra-rater (ICC = .95-.98) and interrater (ICC = .95-.99) reliability.^26^ One of the 6 CCS tests is the Interlimb Coordination Test (ILC2), assessing bilateral UL coordination, in which seated individuals perform alternating anti-phase forearm rotations (i.e., pronation-supination) on their knees for 10s. In the CCS, the ILC2 test of rhythmic bimanual anti-phase movement was chosen since, rather than being driven by a common neural generator as used for in-phase movement, anti-phase movement is driven by more independent pathways and involves stronger interhemispheric coupling between motor regions.^27,28^ Anti-phase movement is less stable than in-phase movement, has higher variability and may involve more compensatory movement.^7,18,29^ Performance and movement quality (i.e.,, presence of compensations) are scored on 0–3 pt subscales for a maximum of 6-pts. Although clinicians can assess UL movement characteristics by observation with moderate to high accuracy,^30^ validity testing is needed.

Thus, this study aimed to determine the construct validity of the ILC2 by assessing interlimb coordination at 2 levels of movement description in healthy individuals and in individuals with stroke using highly accurate motion analysis. According to the COSMIN (COnsensus-based Standards for the selection of health status Measurement INstruments) panel definition, construct validity is the degree to which test scores are consistent with the hypotheses regarding internal relationships of scored items, relationships with other test scores, or differences between relevant groups.^31^ Based on this definition, we compared subjective scores of the total ILC2 and its sub-scales between healthy and stroke groups and within groups to objective kinematic measurements of coordination (i.e., cross correlation coefficient, lag and continuous relative phase; CRP) as well as to scores of clinical assessments at the ICF impairment and activity levels. We hypothesized that coordination and kinematic measures of anti-phase forearm rotations would differ between healthy and stroke participants. Since movement characteristics vary with frequency, we also evaluated movements at 2 different frequencies and matched frequencies between the stroke and healthy groups and hypothesized that higher movement frequencies would lead to a greater disruption in coordination and kinematic measures in the stroke group (Hypothesis 2). Our third hypothesis was that participants with stroke who had greater sensorimotor impairments would have greater disruption in coordination measures.

## Methods

### Participants

Fifteen healthy individuals (controls) and 17 individuals with chronic stroke were recruited between July 2019 and March 2020. Participants with stroke diagnosed ≥3 months previously were included if they were ≥40 years old, had any type/level of stroke severity, could perform isolated supination of ≥30° of the more-affected (MAff) arm, and could understand and follow instructions. Severe cognitive impairments (MoCA < 23),^32^ receptive language impairments, ataxia or apraxia according to chart review were exclusion criteria. For both groups, individuals with musculoskeletal or other injuries interfering with task performance were excluded. Two participants with stroke were excluded due to severe cognitive impairments. Data of 2 controls and 2 participants with stroke were excluded due to technical problems. The final cohort included 13 individuals per group. The study was approved by the Research Ethics Board of the Centre of Interdisciplinary Research in Rehabilitation, and all participants signed approved informed consent forms.

### Procedure

Each individual participated in one 2-hour session that included clinical assessments and kinematic measurements of the ILC2.

### Clinical Assessments

UL motor impairments and activity limitations were evaluated using standardized clinical tests. Impairments were assessed with the Fugl–Meyer Assessment-Upper Limb (FMA-UL)^33^ in the stroke group and the Finger-To-Nose test (FTN)^34^ and ILC2^25^ in both groups. The Activity level Chedoke Arm and Hand Activity Inventory-9^35^ was used in the stroke group and the Box and Block Test (BBT)^36^ was used in both groups.

**ILC2** measures coordination between rotations of both forearms. With forearms supported on their knees, seated participants performed anti-phase rotational movements of both forearms for 10 s. Coordination was scored at performance and movement quality levels, each on scales of 0 (impaired) to 3 (normal) pts for a total of 6-pts (i.e.,, ILC2-total). At the performance level, an ILC2-synchronicity subscale was scored based on anti-phase movement duration without considering the range of motion (ROM) used by forearms, elbows or shoulders (i.e.,, 3: 10 s; 2: 5-10 s; 1: <5 s; 0: unable to perform). At the movement quality level, an ILC2-compensation subscale was based on forearm ROM and arm position relative to the trunk (i.e.,, 3: both elbows maintained close to trunk and full forearm ROM; 2: both elbows maintained close to trunk with partial forearm ROM; 1: only one elbow close to trunk; or 0: neither elbow close to trunk).

**FMA-UL** is a stroke-specific performance-based impairment scale that includes 4 UL elements scored on 3-pt ordinal scales: motor function (66-pts), sensation (12-pts), passive ROM (24-pts), and joint pain (24-pts), for a total of 126-pts. Higher scores indicate less impairment. The FMA-UL has excellent interrater (ICC = .97-.99) and test-retest (ICC = .81-.97) reliability in sub-acute and chronic stroke.^37^ The FMA-UL motor score was strongly correlated with the Action Research Arm Test (ARAT; r = .93) and BBT (r = .92).^37^

**FTN** is a common clinical measure used to assess UL interjoint coordination in which seated individuals alternately touch their nose and a target located at arm’s length and nose height.^38^ For each arm, the final score includes the time to complete 5 movement cycles, 2 performance (i.e., movement smoothness and endpoint accuracy) and 2 movement quality elements (i.e., trunk stability and arm movement), each on scales of 0-3 for a total of 12-pts. Swaine and Sullivan^34^ reported excellent intra-rater (ICC = .97-.99) and interrater reliability (ICC = .91-.92) in individuals with traumatic brain injury. The FTN was strongly correlated with measures of UL performance and dexterity (r = .79-.82).^39^ Rodrigues et al^38^ found that kinematic variables (i.e., shoulder ROM, cross-correlation lag and the ratio between shoulder and elbow movements) explained more than 80% of the variance in the FTN time in post-stroke individuals.

**CAHAI-9** assesses functional ability of the MAff UL during 9 bimanual tasks. Tasks are rated on scales of 1 (total assistance) to 7 (complete independence), for a maximum of 63-pts.^35^ CAHAI-9 has excellent test-retest reliability (ICC = .97) and was strongly correlated with ARAT (r = .94) and Chedoke–McMaster Stroke Assessment (r = .91).

**BBT** measures manual dexterity as the number of blocks transferred from one side of a box to the other in 1 min by each hand. A higher number of transferred cubes indicates better manual dexterity. Raw scores in both groups for each hand were normalized according to normative healthy adult data.^36^ The BBT has excellent test-retest (ICC = .93-.98) and interrater (ICC = .99) reliability in chronic stroke^40^ and was strongly correlated with ARAT (r = .95) and FMA (r = .92).^37^

### Kinematic Assessment

Kinematics of both arms were recorded during performance of the ILC2. To avoid a learning effect, participants practiced until they felt comfortable with the task and the performance stabilized (i.e.,, frequency maintained for ≥10 rotations, 1-6 practice trials). To minimize fatigue, 1-5 min rest periods were allowed between trials.

Participants were comfortably seated on an adjustable chair with feet supported on the floor and the hips and knees flexed to 90°. In the initial position, the trunk was supported by the chair back, and elbows were parallel to the trunk. The right hand dorsum was placed on the right knee and the left palm was placed on the left knee. Participants were instructed to perform continuous anti-phase rotational movements of both forearms by alternately touching their knees with their palms and hand dorsa for 10 s while maintaining their arms relaxed and parallel to the body. Since frequency influences movement stability,^41^ 4 speed conditions were tested to compare kinematics between groups: 2 internally-paced (IP) and 2 externally-paced (EP). In the IP conditions, participants performed the task at a self-paced speed (IP1) and as fast as possible (IP2) for 10 s. For the EP conditions, participants matched metronome pacing set to 1 Hz (EP1) and 1.5 Hz (EP2) for 10 s and tried to maintain the frequency for an additional 10 s after the metronome was stopped. Three trials were recorded per condition.

Movements were recorded (120 Hz) with a wireless electromagnetic tracking system G4 (Polhemus, Vermont), consisting of a 4 in^3^ (10.16 cm^3^) magnetic field generator (source), transmitters (hubs) and 9, 6 degree-of-freedom (DF) sensors. The root mean square static system accuracy is .08 in (.20 cm) for position and .50° for orientation (Polhemus G4, User Manual). Sensors placed on both hands (radial side of second metacarpal heads), mid-forearms, mid-upper arms, superior-lateral acromion processes, and mid-sternum tracked UL and trunk movements.

### Outcome Measures

Primary outcomes were temporal (i.e., cross-correlation, 0 ± 1 and lag, s) and spatio-temporal (CRP, 0-360°) values describing bilateral forearm rotations (Hypotheses 1 and 2). The cross-correlation coefficient was used to measure the temporal coupling between forearm movements, with values near zero indicating that the forearms move independently and highly positive/negative values indicating an in-phase or anti-phase relationship, respectively. The CRP is a spatiotemporal representation of the differences in phase angles of movements of 2 elements. A CRP of 0° indicates an in-phase relationship, and a CRP of 180° (or −180°) indicates an anti-phase relationship. Secondary outcomes for Hypotheses 1 and 2 were the quality of trunk and UL movements (trunk flexion, rotation and side flexion, shoulder flexion, abduction and rotation, and elbow flexion) measured in degrees. For Hypothesis 3, clinical assessments were correlated with primary and secondary outcomes.

For the forearm movements, Akima cubic Hermite interpolation and filtering was done using a 250 ms triangular window moving average. The cross-correlation sequence was normalized so that the autocorrelations at zero lag were equal to 1 according to the following equation 1

(1) $$\mathrm{NC}C_{\left(x,y\right)}\left(m\right)=\frac{CC_{\left(x,y\right)}\left(m\right)}{\sqrt{CC_{\left(x,x\right)}\left(0\right)\times CC_{\left(y,y\right)}\left(0\right)}}=\frac{CC_{\left(x,y\right)}\left(m\right)}{\sqrt{\Sigma x^{2}}\;\times \;\sqrt{\Sigma y^{2}}}$$

where NCC is the normalized cross-correlation, cc is the cross-correlation and *m* is the lag value for *x* and *y* signals corresponding to the left and right forearm rotations.

The lag corresponded to the time of the minimal cross-correlation value (i.e.,, anti-phase). The similarity between supination/pronation movements was assessed by aligning and comparing roll angles of both forearms.

The CRP was used to determine the phase difference between the 2 signals at each time point.^42^ The leading limb was defined as the dominant (Dom) side in controls and as the less-affected (LAff) side in the stroke group. Roll signals of both forearms were centered around 0 and Hilbert transformed to create the analytic angles. Phase angles of each roll signal were calculated by plotting the normalized roll angle and velocity signals for each forearm on the x-y plane. A CRP polar angle was defined between the positive *x*-axis and the line from the origin to the point of interest in the counter-clockwise direction. The normalized CRP value (0-360°) between the 2 signals was calculated by subtracting the phase-angle of the non-leading from the leading limb, and computing the absolute CRP distance from a “perfect” anti-phase coordination (i.e., CRP = 180°).

Joint rotations were calculated in trunk-centered absolute coordinates (*x*, *y*, *z*). Trunk flexion was calculated between the vector in line with the trunk through the mid-sternal marker and the vertical vector through the same marker where the initial position was defined as 0° and positive values indicated trunk flexion. Trunk rotation and side-flexion were calculated from the same marker, where positive values indicated left rotation and left side-flexion, respectively. For shoulder flexion and abduction, 2 vectors were formed: one between the 2 shoulder markers and another from the right or left shoulder marker to the right or left elbow marker projected on the sagittal and transverse planes, respectively. Shoulder rotation was the angle between the planes formed by the shoulder, upper-arm and mid-sternal markers, and the shoulder, upper-arm and forearm markers projected on the coronal plane (*z*, *x*). For all 3 shoulder DFs, the initial arm position was defined as 0° and higher values indicated greater angles. Elbow flexion was defined as the angle between the shoulder-upper-arm vector and the elbow-forearm vector.

### Statistical Analysis

Movement of the Dom and NonDom arms were compared to movements of the LAff and MAff arms of participants with stroke when indicated. Normality of distributions was determined using Shapiro–Wilk tests and homogeneity of variances was determined using Levene’s tests (SPSS 27.0, SPSS Inc., Chicago, IL). Initial significance levels were P < .05, and Bonferroni corrections were applied for multiple comparisons (i.e., Hypothesis 2).

For Hypothesis 1, frequencies were compared between groups with Kruskal–Wallis one-way analyses of variance (ANOVA) and between conditions with Friedman’s tests. Similarity between supination/pronation movements of both forearms was assessed with Pearson correlations at zero lag.

Hypothesis 2 was tested using Generalized Estimating Equation modeling with Bonferroni adjusted P-values for between- and within-subject factors and their interactions (groups: healthy, stroke; conditions: IP1, IP2, EP1, and EP2) to detect differences in coordination and kinematic measures. In addition, to compare coordination (CRP, cross-correlation, and lag) and kinematic variables between groups in IP1 and a matched-speed condition of similar forearm rotation frequencies (i.e.,, IP1 in healthy and IP2 in stroke), one-way ANOVA and Kruskal–Wallis tests were used.

For Hypothesis 3, scores of the ILC2 in the stroke group were correlated with: (1) coordination measures for IP1 and IP2; (2) kinematic variables for IP1 and IP2; and (3) clinical assessments (i.e., FMA, FTN, CAHAI-9, BBT) using Spearman rank-order correlations (rho) or Chi-square correlations. Spearman rho values <.3, −.3-.59 and ≥.6 were considered as weak, moderate, and strong, respectively.^43^ Effect sizes were calculated using Cohen’s d.

## Results

Participants ranged in age from 40 to 89 years with no difference between groups (Table 1). Stroke participants had sustained a unilateral ischemic or hemorrhagic stroke, 16–68 months earlier, had mild-to-moderate UL sensorimotor impairment and activity limitations and no marked cognitive deficits (Table 1). No participant had marked tactile or proprioceptive deficits based on FMA-sensation scores.

**Table 1.** Demographic Data of Participants and Scores of Clinical Assessments. Values are mean (SD) or number (%).

	Healthy n = 13	Stroke n = 13
Sex - female (%)	6 (46.2)	4 (36.4)
Age, yr	68.5 (14.3)	65.1 (12.3)
Hand dominance - right (%)	9 (69.2)	12 (92.3)
Time since stroke, mo		37.8 (16.4)
Type of stroke - ischemic (%)		8 (61.5)
Side of hemiparesis - right (%)		6 (46.2)
ILC2 total score*, pt; max score = 6 pt	5.6 (.5)	4.6 (.9)
FMA-UL, motor score, pt; max score = 66 pt		58.3 (6.0)
FMA-UL, sensation, pt; max score = 12 pt		11.0 (1.4)
MoCA, otl max score = 30 pt		26.3 (1.9)
BBT, total number of cubes transferred*	113.1 (15.6)	92.8 (22.1)
BBT, normalized score*%	82.5 (10.8)	65.3 (12.9)
FTN total score*, pt; max score = 24 pt	23.9 (.3)	22.2 (1.8)
FTN time, dominant/less affected, s	4.4 (.9)	5.6 (1.3)
FTN time, non-dominant/more affected, s	4.6 (.9)	7.7 (3.4)
CAHAI, pt; max score = 63 pt		56.0 (10.2)

*Sum of both arms.

Abbreviation: BBT, Box and Blocks Test; CAHAI, Chedoke Arm and Hand Activity Inventory; FMA-UL, Fugl Meyer Assessment for the Upper Limb; FTN, Finger-To-Nose Test; ILC2, Interlimb Coordination of upper limbs; MoCA, Montreal Cognitive Assessment.

### Coordination Measures

**Figure 1.**
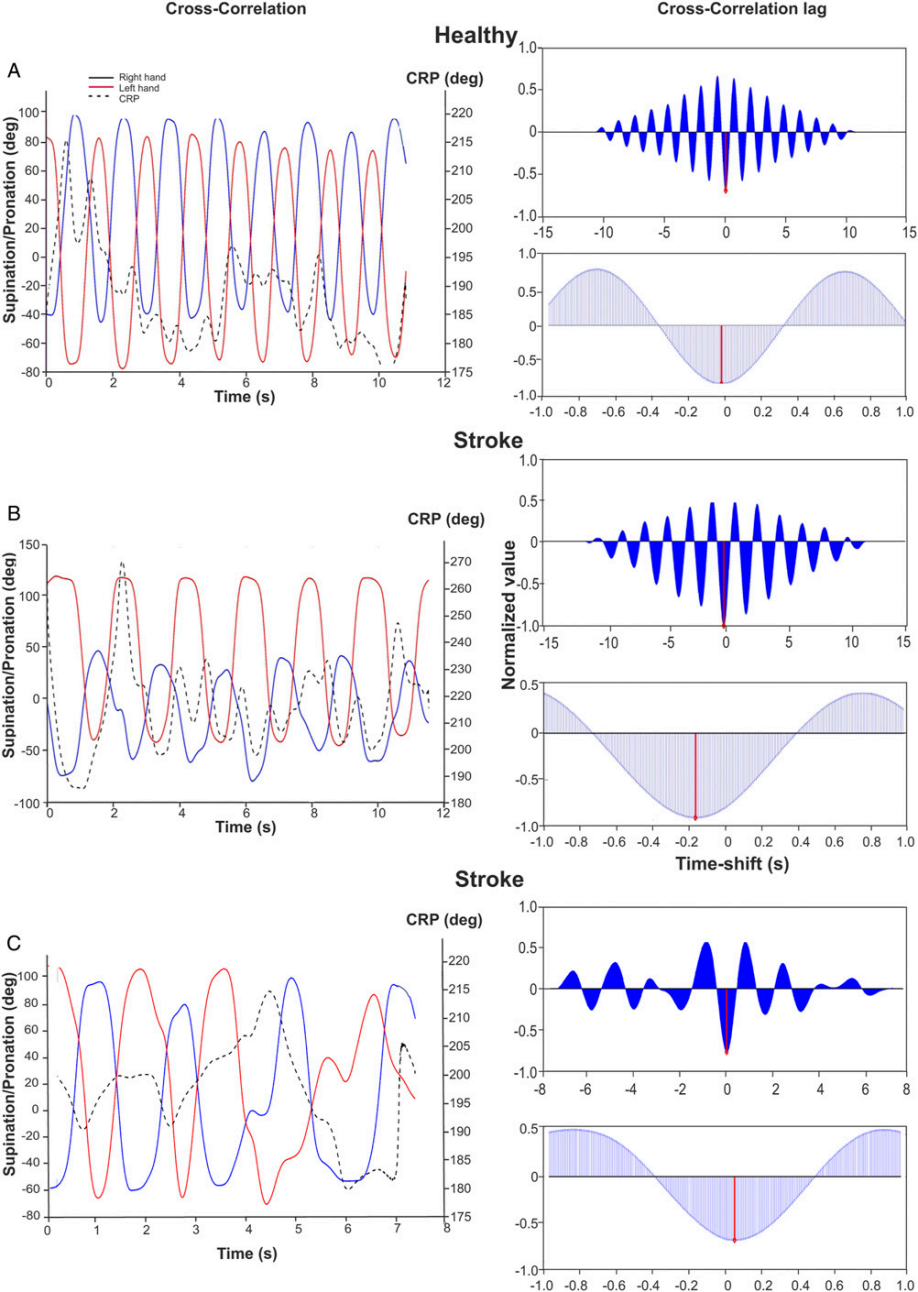
Forearm movements, continuous relative phase (CRP), and cross-correlogram in 1 representative healthy subject (a) and 2 representative subjects with stroke (b, c) for the self-paced condition (Internally-paced, IP1). Left panels: Right and left forearm rotational movements in the horizontal plane (pro-supination; solid lines) and CRP (dashed line). Right panels: Top: Cross-correlogram for the whole trial duration used for calculation of the lag between the forearm movements. Bottom: The anti-phase lag for the cycle is the point corresponding to the minimal cross-correlation. Cross-correlation values are expressed as normalized values.

**Figure 2.**
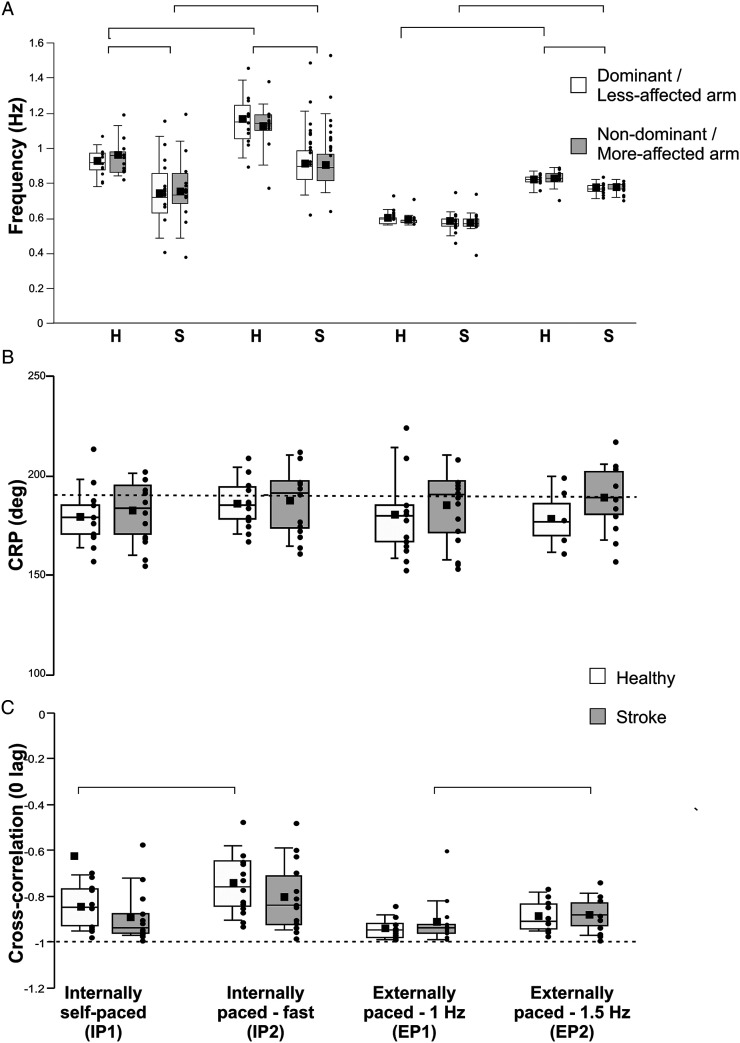
Median, mean, first quartile, third quartile, minimum, and maximum values of temporal and spatial measures of coordination between groups (healthy, stroke) and conditions (IP1: Internally self-paced, IP2: Internally-paced fast, EP1: Externally paced at 1 Hz, EP2: Externally-paced at 1.5 Hz). Data shown for each arm in A and for both arms in B and C. Significance indicated by horizontal bars. In A, horizontal bars indicate significant differences for both arms. CRP: continuous relative phase; H: healthy; S: stroke.

Compared to healthy participants, stroke participants moved both forearms ∼23% slower in IP1 (LAff: H = 6.989, P = .008, d = 1.007; MAff: H = 8.123, P = .004, d = 1.066), ∼22% slower in IP2 (LAff: H = 7.118, P = .008, d = 1.130; MAff: H = 5.815, P = .016, d = .983) and 5–6% slower in EP2 (LAff: F_1,24_ = 7.084, P = .014, d = 1.138; MAff: H = 9.243, P = .002, d = 1.019; Figures 1 and 2(a)). Forearm rotation frequencies were similar between groups and arms in EP1 and the matched-speed condition.

When asked to move faster (IP2), forearm movement frequency increased by 19–26% in controls (χ^2^(1) = 6.231, P = .013; Dom: d = −1.631, NonDom: d = −1.039), and by 22–25% in participants with stroke (χ^2^(1) = 13.00, P < .001; LAff: d = −.713, MAff: d = −.622). Between EP conditions, forearm movement frequency increased by 37–51% in controls (χ^2^(1) = 5.33, P = .02; Dom: d = −5.100, NonDom: d = −4.306) and by ∼33% in stroke participants (χ^2^(1) = 9.308, P = .002; LAff: d = −2.917, MAff: d = −2.787, Figure 2(a)).

In both IP and EP conditions, CRP tended to be closer to 180° when participants moved their forearms at slower speeds with no differences between groups or conditions (Figure 2(b)). The cross-correlation between forearm movements was less negative when frequency was increased (group x condition effect: Wald χ^2^(1,3) = 14.192, P = .003). This effect was present in controls for the self-paced condition (P = .005, d = −.578) and in the stroke participants for the EP condition (P = .000, d = −.826). The highest temporal relationship (cross-correlation closer to −1) occurred in controls in EP1 (cross-correlation coefficient = −.94 ± .04, Figure 2(c)). Cross-correlation lags did not differ between conditions with averages ranging from .03 to .07 s.

### Kinematics

For all conditions, participants with stroke used more shoulder abduction of the LAff (Wald χ^2^(1,3) = 3.829, P = .050, d = 2.795) and MAff (Wald χ^2^(1,3) = 5.555, P = .018, d = 4.486) arms compared to Dom and NonDom arms of controls (Table 2).

**Table 2.** Secondary Outcome Measures by Condition.

	Healthy				Stroke			
	IP1	IP2	EP1	EP2	IP1	IP2	EP1	EP2
Trunk flexion**	1.4 (1.2)	2.7 (1.2)**	2.6 (1.4)	2.2 (1.3)	3.9 (3.5)	3.8 (3.7)	3.6 (3.5)	2.6 (2.9)
Trunk rotation**	1.0 (.7)	2.8 (2.9)	2.4 (3.2)	1.6 (1.7)	2.3 (1.4)	2.8 (1.6)	2.7 (1.9)	3.2 (1.6)**
Trunk side flexion	1.3 (1.0)	2.0 (1.9)	1.6 (.9)	1.9 (1.3)	12.0 (1.6)	2.5 (1.9)	2.3 (2.1)	2.9 (2.2)
Shoulder flexion Dom/LAff**	19.0 (6.9)	14.5 (8.7)	15.6 (9.3)	14.5** (9.3)	13.3 (12.0)	14.2 (11.5)	13.7 (11.7)	18.4 (10.1)
Shoulder flexion NonDom/MAff **	16.8 (10.2)	12.6** (11.0)	14.6 (13.1)	14.4 (13.1)	17.8 (13.0)	19.8 (12.7)	18.5 (12.9)	21.9 (12.1)
Shoulder abduction* Dom/LAff	5.5 (5.4)	6.7 (9.0)	4.6 (6.9)	6.0 (8.2)	9.5 (9.8)	11.2* (9.8)	9.8 (9.8)	15.5 (5.5)
Shoulder abduction* NonDom/MAff	3.7 (5.5)	5.9 (7.0)	3.6 (7.1)	3.3 (6.0)	9.2 (9.5)	10.9* (9.8)	9.6 (9.0)	12.9 (8.1)
Shoulder rotation Dom/LAff	8.8 (6.2)	5.7 (5.3)	7.1 (4.3)	6.8 (3.6)	7.0 (4.4)	6.0 (4.6)	8.1 (5.0)	8.0 (4.8)
Shoulder rotation NonDom/MAff**	9.4 (5.9)	4.9 (4.3)**	6.9 (5.0)	5.8 (4.9)	6.6 (4.5)	6.8 (4.3)	6.8 (5.4)	6.4 (4.7)
Elbow flexion - Dom/LAff	8.9 (5.2)	11.2 (9.3)	8.3 (7.5)	10.7 (9.4)	14.3 (11.0)	14.3 (10.4)	13.6 (11.0)	13.7 (7.9)
Elbow flexion Non-Dom/MAff	12.7 (8.3)	15.3 (12.7)	12.6 (8.1)	16.1 (13.3)	14.3 (9.1)	12.6 (8.7)	13.1 (9.5)	11.6 (8.9)

*significant group effect; ** significant group x condition effect.

Abbreviation: Dom, dominant; NonDom, non-dominant; LAff, Less affected; MAff, More affected.

Range of motion of trunk, shoulder and elbow movements for the internally-paced speed conditions (self-paced: IP1; fast self-paced: IP2) and the externally paced (EP) at 1 Hz (EP1) and at 1.5 Hz (EP2) for healthy and stroke groups. Values are expressed in degrees (mean SD).

When moving faster in the IP condition, healthy subjects used more trunk flexion (Wald χ^2^(1,3) = 9.666, P = .022, d = 1.045) and less shoulder flexion (Wald χ^2^(1,3) = 13.630, P = .003, d = −3.911) and shoulder rotation (Wald χ^2^(1,3) = 12.236, P = .007, d = −.957) in the NonDom arm. In the stroke group, there were no differences in kinematics between IP1 and IP2.

For the EP condition, participants with stroke used more trunk rotation (Wald χ^2^(1,3) = 8.062, P = .045, d = .290) and tended to increase shoulder flexion in both arms, while control subjects used slightly less shoulder flexion in the Dom arm (Wald χ^2^(1,3) = 11.524, P = .009, d = −.121).

### Between-group differences—matched-speed condition

**Figure 3.**
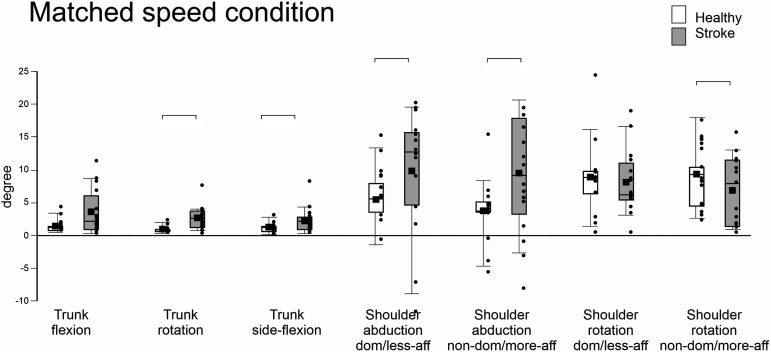
Median, mean, first quartile, third quartile, minimum, and maximum values of ranges of trunk and shoulder movements in healthy and stroke participants in the matched-speed condition (Healthy: Internally-Paced 1, IP1; Stroke: Internally-Paced 2, IP2).

In the matched-speed conditions, all primary outcome measures (i.e., CRP, cross-correlation and lag) were similar between groups. Compared to healthy subjects, participants with stroke used 197% more trunk rotation (H = 12.185, P < .001, d = −1.505), 118% more trunk side-flexion (F_1,24_ = 6.281, P = .021, d = −.803), and 115% and 235% greater shoulder abduction of the LAff (H = 5.215, P = .022, d = −.714) and MAff arms (F_1,24_ = 7.773, P = .01, d = −.900), respectively (Figure 3). However, they used 72% less shoulder rotation of the MAff arm (F_1,24_ = 5.234, P = .031, d = .494) compared to controls. Trunk flexion, shoulder flexion (both arms), shoulder rotation (LAff arm), and elbow flexion (both arms) did not differ between groups (Table 3, Figure 3).

**Table 3.** Secondary Outcome Measures.

	Self-Paced Speed		Matched-Speed	
	Healthy (IP1)	Stroke (IP1)	Healthy (IP1)	Stroke (IP2)
Trunk flexion	1.4 (1.3)	2.1 (.9–5.9)^a^	1.4 (1.3)	3.0 (3.2)
Trunk rotation	.9 (.5–1.1)^a^	2.7 (1.2–3.1)^a^ *	.9 (.5–1.1)^a^	2.9 (1.6)*
Trunk side flexion	1.3 (1.1)	2.1 (1.7)	1.3 (1.1)	2.8 (1.9)*
Shoulder flexion – Dom/LAff	19.1 (7.2)	13.4 (12.8)	19.1 (7.2)	14.6 (12.1)
Shoulder flexion – NonDom/MAff	18.9 (14.5–23.0)^a^	17.7 (12.5)	18.9 (14.5–23.0)^a^	19.6 (11.6)
Shoulder abduction - Dom/LAff	5.5 (5.6)	10.2 (7.9)	5.5 (5.6)	11.9 (7.9)*
Shoulder abduction - NonDom/MAff	3.7 (5.8)	10.2 (9.8)*	3.7 (5.8)	12.4 (9.6)*
Shoulder rotation - Dom/LAff	−8.0 (7.3)	−7.1 (4.5)	−8.0 (7.3)	−4.6 (5.8)
Shoulder rotation - NonDom/MAff	−8.8 (6.8)	−4.7 (5.9)	−8.8 (6.8)	−2.5 (7.3)*
Elbow flexion - Dom/LAff	7.8 (6.7)	12.1 (14.4)	7.8 (6.7)	12.9 (12.9)
Elbow flexion - NonDom/MAff	11.3 (10.1)	10.7 (13.9)	11.3 (10.1)	10.4 (11.9)

^a^median (IQR).

^a^median (IQR); *significant difference between groups.

Abbreviation: Dom, dominant; NonDom, non-dominant; LAff, Less affected; MAff, More affected.

Range of motion of trunk, shoulder and elbow movements for the self-paced speed condition (Internally-paced 1, IP1) for both groups and the matched-speed condition (Internally-paced, IP1 in the healthy group and Internally-paced 2, IP2) in the stroke group. Values are expressed in degrees (mean SD).

### Relationship Between UL Sensorimotor Impairment and Coordination

ILC2 sub-scores and total score were not correlated with coordination measures for both IP conditions in controls. However, in the stroke group, in IP2 but not IP1, ILC2-synchronicity and ILC2-total scores were related to temporal coordination measures. A higher ILC2-synchronicity score was related to a shorter lag (χ^2^(1) = 13.074; P = .011, d = 5.211) and a higher ILC2-total score was related to a smaller cross-correlation coefficient (i.e., closer to 0; χ^2^(1) = 12.567, P = .05, d = 4.437). In the stroke group, the ILC2-total score was related to secondary outcome measures (i.e., kinematics) for both IP conditions. Greater shoulder rotation of the MAff arm was related to higher ILC2-total scores in IP1 (χ^2^ = 12.740, P = .047, d = 4.786) and IP2 (χ^2^ = 13.186, P = .04, d = 5.392). ILC2 sub-scores and total scores were not correlated with clinical scores in either group.

## Discussion

We found that ILC2 is a valid measure of UL interlimb coordination. Healthy participants had near maximal ILC2 scores and high temporal and spatial coordination indices. However, participants with stroke had lower ILC2 scores and used trunk and shoulder compensations to perform the task. ILC2 scores distinguished between healthy participants and participants with chronic stroke.

### Effect of Speed and Pacing on Interlimb Coordination

In both IP conditions, movement frequency was higher in healthy compared to stroke participants. In both groups, increasing the movement frequency led to lower cross-correlations while CRP values were similar. Interestingly, stroke participants had the highest cross-correlation values at the slow self-paced speed (IP1). This suggests that limiting the movement speed was a necessary condition of better coordination in this group,^44^ reminiscent of the speed-accuracy trade-off.^45^ The difficulty of producing self-paced fast movement in the stroke group may be related to decreased facilitation in interhemispheric inhibitory and facilitatory circuits.^11^

External pacing resulted in better matching of frequencies between groups at both speeds and similar cross-correlation values (Figure 2). Notably, when movement speed was externally paced, increasing the speed had the same effect as IP speed increases. This is consistent with previous suggestions that external auditory pacing improves intra- and inter-limb coordination in healthy individuals as well as in individuals with stroke and other neurological deficits. Thaut and colleagues^46^ examined how rhythmic cueing affected spatiotemporal control of repeated reaches in people with chronic stroke. Timing and movement trajectories were less variable, movements were smoother and elbow ROM increased with external pacing compared to self-paced movements. The beneficial effect of auditory cueing on motor performance may be explained by entrainment related to neural connectivity between auditory rhythm and movement areas at cortical, subcortical, and spinal levels.^47,48^ For example, auditory stimuli during motor task performance facilitated neural activity in premotor cortex, insula, supplementary motor area, cerebellum, and basal ganglia. This process may influence temporal coupling to enhance motor performance.^49^

### Effect of Speed on Movement Patterns

Movement frequency is considered a dynamical coordination control parameter.^18,50,51^ The temporal relationship between rhythmical bimanual movements emerges from the properties of dynamical oscillators. In such movements, the system constrains the redundant DFs by organizing itself as a nonlinear system of coupled oscillators. The CRP, a collective variable describing this relationship and its stability, depends on the initial state of the control parameter, that is, frequency. When movement frequency is increased, the stability of arm movements is undermined, and if it exceeds a critical threshold, a spontaneous phase transition to a more stable in-phase pattern occurs.^52,53^

Participants with stroke preserved bimanual coordination despite the increase in speed, but did so by using more compensatory movements. The similarity in coordination measures between groups may reflect the tendency of the system to preserve the temporal stability of forearm movements, even at the expense of using compensatory movements of other body segments like the trunk and shoulders. This is consistent with the principle of motor equivalence, in which the system uses the available DFs to stabilize the task performance.^16,54^ The ability to use compensatory, motor equivalent actions results from the redundant number of muscles and joints of the motor system.^55^ Motor redundancy has been described as a problem for the motor system to solve in order to choose optimal solutions to a motor task. However, the abundant possible solutions also allow the motor system to be flexible and select different solutions while maintaining performance accuracy.^55^ This ability is relevant for the performance of daily activities in which task demands and the environment vary.^8^ In this study, participants with stroke used a different set of joint rotations, or motor compensatory movements to perform the task. These compensations may result from symmetry-breaking forces^8,56^ due to impaired interjoint coordination^57^ or differences in biomechanical properties such as muscle strength or spasticity.^58^ The use of compensatory movements in the stroke groups, with similar coordination measures to controls suggests that these compensations assisted participants with stroke to stabilize their performance and maintain the anti-phase forearm movements.

### Relationship Between ILC2 Scores, Coordination Measures, and Compensatory Movements

ILC2 total score and synchronicity sub-scores of stroke participants were related to temporal coordination measures (i.e., lag, cross-correlation), but not to spatiotemporal coordination (i.e., CRP). In the ILC2, the performance level is assessed temporally as the ability to move both forearms synchronously for 10 s. Relationships between ILC2 and temporal coordination measures in the stroke group were found for IP2, but not IP1. Increasing the frequency may have challenged the temporal stability of the movement, even though no phase-transition occurred, such that it was reflected in the ILC2 score. The presence of these relationships in the stroke but not the healthy group may be due to the high performance level attained by healthy participants on the ILC2. Thus, the ILC2 may be a good measurement of temporal coupling of forearm movements in stroke populations, but may not be sensitive enough to detect differences in healthy participants. Furthermore, relationships between ILC2-total score and greater shoulder rotation of the MAff arm, but not with other kinematic variables suggests that ILC2 is sensitive to shoulder rotation compensatory movements, but may not be sensitive enough to detect excessive trunk and elbow movements.

### Relationship Between ILC2 and Clinical Assessments

No relationships between ILC2 and other clinical assessments were found, since these assessments do not capture temporal and spatial aspects of interlimb coordination. Even though the assessments used have good measurement properties and have been used extensively in neurological research and clinical practice, scoring is based on the performance of only the MAff limb, rather than both limbs as in the ILC2. Although rehabilitation interventions are often based on the assumption that improvements in the performance of the MAff UL will lead to an improvement in bimanual performance, this is not supported by research findings. In an action requiring bimanual coordination, the redundant DFs (e.g., shoulder and elbow rotations) are constrained such that the ULs are temporally and/or spatially coupled and act as a single unit. Therefore, it cannot be assumed that performance of one limb will reflect the performance of both limbs acting together.^7,18^ Although the CAHAI is aimed at assessing the functional ability of the MAff arm in bimanual motor tasks, scoring is only based on the contribution of the MAff arm,^35^ and therefore does not reflect bimanual function. Moreover, scoring focuses on task completion and endpoint characteristics. Rather than being directly identified, motor compensations are only assumed to contribute to slower movements or performance difficulties resulting in partial scores.^23^ Thus current assessments are limited in their ability to track changes over time and distinguish recovery from compensations.

### Limitations

Although our sample size was relatively small, the high obtained power suggests that a larger sample would not have significantly affected the results. The construct validity of ILC2 was only evaluated in individuals with chronic stroke and may not generalize to patients in earlier recovery stages. Interlimb coordination deficits occurring in other neurological conditions such as Parkinson’s disease, multiple sclerosis, and ataxia may have different characteristics. It is unknown whether ILC2 is able to discriminate between different neurological populations. Individuals with apraxia, ataxia, severe cognitive impairments, or severe language impairments were excluded from this study, also limiting results generalizability. The ILC2 measures symmetrical movements of both limbs, while asymmetrical movements are often required for functional motor tasks (e.g., tying shoelaces).^58^ This limits the generalizability of the results to all bimanual actions. Finally, the ILC2 measures anti-phase movements requiring suppression of bilateral synchronization and stronger interhemispheric coupling of motor regions than in-phase movements.^27,28^ Thus, conclusions about the use of compensatory movements used for the ILC2 cannot be generalized to the production of synchronous in-phase movement. Further tests of validity may be done by comparisons between ILC2 movement patterns and those of a unilateral task and/or a bilateral in-phase task.

## Conclusion

Our results support the hypothesis that the ILC2, one of 6 tests of the CCS,^25,26^ is a valid assessment of UL interlimb coordination that is able to distinguish recovery from compensation by describing movement at both the performance and the movement quality levels. Moreover, it is the only clinical coordination measure assessing movements of both ULs as a single unit rather than each individually. This test can be used to gain a better understanding of the relationship between deficits in UL interlimb coordination and functional recovery. Furthermore, it can guide clinicians in tailoring intervention plans and goals for individuals with chronic stroke and follow-up on their condition. Future studies may assess validity of the ILC2 in acute and sub-acute patients with stroke or other neurological conditions, such as Parkinson’s disease, multiple sclerosis, or traumatic brain injury.
